# Identification of Binding Partners of Deafness-Related Protein PDZD7

**DOI:** 10.1155/2018/2062346

**Published:** 2018-03-28

**Authors:** Haibo Du, Rui Ren, Panpan Chen, Zhigang Xu, Yanfei Wang

**Affiliations:** ^1^Shenzhen Research Institute of Shandong University, Shenzhen, Guangdong 518057, China; ^2^Shandong Provincial Key Laboratory of Animal Cells and Developmental Biology, School of Life Sciences, Shandong University, Jinan, Shandong 250100, China; ^3^Co-Innovation Center of Cell Biology, Shandong Normal University, Jinan, Shandong 250014, China

## Abstract

*PDZD7* is an important deafness gene, whose mutations are associated with syndromic and nonsyndromic hearing loss. PDZD7 contains multiple PDZ domains that are essential for organizing various proteins into protein complex. Several PDZD7-binding proteins have been identified, including usherin, ADGRV1, whirlin, harmonin, SANS, and MYO7A, all belonging to USH proteins. Here, we report the identification of novel PDZD7-binding partners through yeast two-hybrid screening using the first two PDZ domains of PDZD7 as bait. Eleven proteins were identified, most of which have not been reported as PDZD7-binding partners before. Among the identified proteins, ADGRV1, gelsolin, and *β*-catenin have been shown to play important roles in hearing, whereas the functions of other proteins in the inner ear remain elusive. We confirmed the expression of one candidate PDZD7-binding protein, CADM1, in the mouse inner ear and evaluated the auditory function of *Cadm1* knockout mice by performing auditory brainstem response (ABR) measurement. Unexpectedly, *Cadm1* knockout mice show normal hearing threshold, which might be explained by the possible compensation by its homologs that are also expressed in the inner ear. Taken together, our work identified several novel PDZD7-binding proteins, which will help us to further understand the role of PDZD7 in hearing transduction.

## 1. Introduction

Usher syndrome (USH) is the most frequent form of inherited sensory deaf-blindness that is characterized by hearing loss and vision defect [1, 2]. According to the severity of hearing loss as well as the presence or absence of balancing problems, USH is clinically classified into three subtypes, namely, USH1, USH2, and USH3, with USH1 as the most severe one. At present, ten genes have been associated with USH, including *MYO7A*, *USH1C*, *CDH23*, *PCDH15*, *USH1G*, *CIB2*, *USH2A*, *ADGRV1*, *WHRN*, and *CLRN-1* [3–15]. Mutations of *USH* genes are also responsible for nonsyndromic hearing loss. USH proteins have been shown to interact with one another and form multiprotein complexes and play important roles in the development, maintenance, and function of stereocilia and synapses in the inner ear sensory hair cells [16].

Recently, *PDZD7* was suggested to be a USH modifier and a contributor to digenic USH [17]. Meanwhile, mutations in human *PDZD7* gene are also associated with nonsyndromic hearing loss DFNB57 [18–20]. Similar to harmonin (USH1C) and whirlin (USH2D), full-length PDZD7 contains three PDZ domains, a harmonin-N like (HNL) domain, and a proline-rich (PR) region. Shorter PDZD7 isoforms containing the first two PDZ domains were also detected in the inner ear [17, 18, 21]. In mice, loss of PDZD7 was shown to result in stereocilia disorganization as well as mechanotransduction deficits [21].

As a PDZ domain-containing scaffold protein, PDZD7 plays important roles in organizing protein complex. PDZD7 has been shown to bind the three known USH2 proteins usherin (USH2A), ADGRV1 (USH2C), and whirlin (USH2D), forming the so-called ankle-link complex at the ankle region of hair cell stereocilia [17, 21–23]. In *Pdzd7* knockout mice, the localization of the three USH2 proteins at the ankle links was interrupted, suggesting that PDZD7 plays a pivotal role in organizing the ankle-link complex [21]. Moreover, PDZD7 was also shown to interact with USH1 proteins MYO7A (USH1B), harmonin (USH1C), and SANS (USH1G) [18, 24, 25].

At present, little is known about other non-USH PDZD7-binding partners. In the present work, yeast two-hybrid screening was performed using the first two PDZ domains as bait to identify new PDZD7-binding partners that are expressed in the inner ear. Identification of PDZD7-binding proteins will help us to further understand the role of PDZD7 in hearing transduction.

## 2. Materials and Methods

### 2.1. DNA Constructs

Mouse cDNA encoding PDZD7 short isoform (amino acids 1–557) was inserted into pBD-GAL4 Cam vector (Stratagene) to express the bait protein for yeast two-hybrid screen. The same cDNA was inserted into pmCherry-N1 or pMYC-C2 (modified pEGFP-C2 with EGFP-coding sequence replaced by Myc-coding sequence) to express PDZD7-mCherry or Myc-PDZD7 fusion protein. Full-length cDNAs encoding mouse *β*-catenin and CADM1, as well as cDNA encoding chicken AMOT (amino acids 311–910), were inserted into pEGFP-C2 to express EGFP-fusion proteins.

### 2.2. Yeast Two-Hybrid Screen

The yeast two-hybrid screen was performed as previously described [26–28]. Briefly, yeast strain AH109 (Clontech) was sequentially transformed with the bait plasmid and a chicken cochlear cDNA library in the HybriZAP two-hybrid vector [29]. A total of 2.4 × 10^6^ transformants were screened using *HIS3* as the primary reporter gene with the presence of 2.5 mM of 3-amino-1,2,4-triazole (3-AT). The positive colonies were further examined using two other reporter genes *ADE2* and *lacZ*. The prey vectors in triple-positive yeast colonies were recovered, and the sequence of cDNA inserts was determined by sequencing.

### 2.3. Colocalization Assay

COS-7 cells were grown on gelatin-coated glass cover slips and transfected with vectors that express target proteins fused to EGFP or mCherry. Twenty-four hours after transfection, cells were fixed with 4% paraformaldehyde (PFA) in PBS for 15 minutes, then permeabilized and blocked with PBT1 (0.1% Triton X-100, 1% BSA, 5% heat-inactivated donkey serum in PBS, pH 7.3) for 30 minutes. For nuclei staining, cells were incubated with DAPI (Gen-View Scientific Inc.) for 15 minutes, then mounted in glycerol/PBS (1 : 1). The subcellular localization of target proteins was examined with a confocal microscope (LSM 700, Zeiss).

### 2.4. Coimmunoprecipitation (co-IP) and Western Blot

HEK293T cells were transfected with vectors that express target proteins fused to EGFP or Myc epitope. Twenty-four hours after transfection, cells were washed with PBS and lysed in ice-cold lysis buffer consisting of 150 mM NaCl, 50 mM Tris at pH 7.5, 1% (vol/vol) Triton X-100, 1 mM PMSF, and 1x protease inhibitor cocktail (Roche). After centrifugation at 4°C, the supernatant was incubated with immobilized anti-Myc antibody (Sigma-Aldrich, Cat. number E6654) at 4°C for 2 hours. Immunoprecipitated proteins were separated by polyacrylamide gel electrophoresis (PAGE), then transferred to PVDF membrane. After blocking in PBS containing 5% BSA and 0.1% Tween-20, the membrane was incubated with anti-Myc (Abmart, Cat. number M20002) or anti-GFP (Abmart, Cat. number M20004) antibody at 4°C overnight, followed by incubation with HRP-conjugated secondary antibody (Bio-Rad, Cat. number 170-6516) at room temperature for an hour. The signals were detected with the ECL system (Cell Signaling Technology, Danvers, MA).

### 2.5. Reverse Transcription-Polymerase Chain Reaction (RT-PCR)

Total RNA of different tissues was extracted using RNeasy Micro Kits (Qiagen) according to the manufacturer's protocol. Reverse transcription (RT) was carried out at 42°C for 1 hour in a 20 *μ*l reaction mixture containing 1 *μ*g of total RNA, 10 pmol of oligo-dT, and 200 units of SuperScript III reverse transcriptase (Invitrogen). Polymerase chain reaction (PCR) was performed using the cDNA as template with the following primers: *Cadm1*: forward primer CGA CAT GGC GAG TGC TGT, reverse primer CCG AAT GAG CCT TTC CCA CT (986 bp); *Cadm2*: forward primer GGC TGC TTC AAA AAG TAA AGT CA, reverse primer GCT GCT AAC GGT GAA GGT CT (523 bp); *Cadm3*: forward primer GCC AAG TCC CTT GTC ACT GT, reverse primer CGC CTT CTG CGT TGA TGA TG (799 bp); *Cadm4*: forward primer TGA AGG ACG AGC GAT TCC AG, reverse primer GTC AGC ACC AGA GTG TCT CC (517 bp); *Necl5*: forward primer TCA CCC TCC TGG ACG AAT CT, reverse primer TGA CAA CGT GGA ATT CGG CA (871 bp); and *β-actin*: forward primer CTC CAT CCT GGC CTC GCT GT, reverse primer GCT GTC ACC TTC ACC GTT CC (268 bp). To obtain the optimal sensitivity and specificity, cycle lengths for different PCR reaction sets were adjusted between 23 and 38 cycles, and annealing temperatures were adjusted between 56 and 64°C. The PCR products were separated by electrophoresis on agarose gel.

### 2.6. Quantitative Real-Time PCR (Q-PCR)

Q-PCR was carried out using SYBR® Premix Ex Taq™ system (Perfect Real Time, Takara) according to the manufacturer's protocol. Amplification and detection were run in a Sequence Detection System SLA-3296 (Bio-Rad) in triplicate with an initial cycle of 95°C for 10 seconds followed by 40 cycles of 95°C for 5 seconds, 60°C for 30 seconds, and 72°C for 20 seconds. Negative control samples (without template) were processed in the same way. The specificity of the amplifications was verified by melting curve analysis. The sequences of primers are as follows: *Cadm1*: forward primer GTG ATC CAG CTC CTG AAC CC, reverse primer CGT GTA GAG CTG GCA GAA GT and *β-actin*: forward primer CTC CAT CCT GGC CTC GCT GT, reverse primer GCT GTC ACC TTC ACC GTT CC. Relative quantization of *Cadm1* expression normalized to *β-actin* was calculated according to the 2^−ΔΔ^CT method.

### 2.7. X-Gal Staining of Mouse Inner Ear

Mouse inner ear temporal bones were dissected and fixed with 4% PFA containing 2 mM MgCl_2_, 5 mM EGTA, and 0.02% NP-40 at 4°C overnight. After rinsing three times with washing buffer (0.1 M PBS, 2 mM MgCl_2_, 0.01% NP-40, and 0.01% sodium deoxycholate), the samples were incubated with staining buffer (0.1 M PBS, 5 mM K_3_[Fe(CN)_6_], 5 mM K_4_[Fe(CN)_6_], 2 mM MgCl_2_, 1 mg/ml X-gal, and 0.01% NP-40) at 37°C overnight. The samples were washed three times with PBS, then the basilar membranes together with the modiolus were dissected out and imaged with a light microscope (Nikon YS100, Japan).

### 2.8. Animal Maintenance and Auditory Brainstem Response (ABR) Measurement

*Cadm1* knockout mice (number RBRC04063) were obtained from RIKEN BioResource Center. Generation and characterization of *Cadm1* knockout mice have been described elsewhere [30, 31]. All animal experiments were approved by the Ethics Committee of Shandong University School of Life Sciences and conducted accordingly. For ABR measurement, mice were anesthetized with 5% chloral hydrate (0.5 ml/100 g body weight). Electrodes were inserted subcutaneously at the vertex, pinna, and near the tail. A RZ6 workstation and BioSig software (Tucker Davis Technologies Inc.) were used for the stimulus generation, presentation, ABR acquisition, and data management. Specific acoustic stimuli were generated using high-frequency transducers, and ABR thresholds were obtained by reducing the stimulus intensity in 10 dB SPL steps to identify the lowest intensity at which all ABR waves were detectable. For noise exposure, mice were exposed to 2–8 kHz noise at 96 dB SPL (Crown, CD i1000) for 2 hours, and ABR thresholds were measured preexposure and at various postexposure time points. For each genotype, at least three animals were used, and data were shown as means ± standard errors. Student's *t*-test was used for statistical analysis, and *p* < 0.05 was considered statistically significant.

## 3. Results

### 3.1. Identification of Potential PDZD7-Binding Partners through Yeast Two-Hybrid Screening

In order to identify new PDZD7-binding partners, we performed yeast two-hybrid screening of a chicken cochlear cDNA library using PDZD7 short isoform as bait. This isoform contains the first two PDZ domains of PDZD7. Around thirty positive clones were obtained that activate all the three reporter genes, representing eleven candidate PDZD7-binding proteins (Table 1). Among the proteins identified, ADGRV1 (USH2C) is a known PDZD7-binding partner, whereas the interactions between PDZD7 and the other proteins have not been reported. The most frequently encountered two proteins are *β*-catenin and ADGRV1, both of which contain a type I PDZ-binding interface (PBI) at their C-termini. Six candidate PDZD7-binding proteins (gelsolin, TRIM35, CADM1, AMOT, Golgin45, and Numb) contain a type II PBI at their C-termini. Three candidates (KCTD10, CCDC27, and TRIP11) do not have a predictable C-terminal PBI.

**Figure 1:**
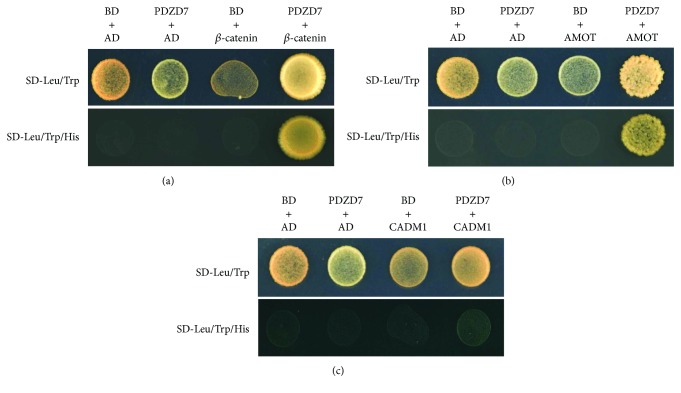
Verification of protein interactions by performing yeast two-hybrid experiments. AH109 yeast cells containing either Gal4 BD/Gal4 AD, Gal4 BD-PDZD7/Gal4 AD, Gal4 BD/Gal4 AD-*β*-catenin, Gal4 BD/Gal4 AD-AMOT, Gal4 BD/Gal4 AD-CADM1, Gal4 BD-PDZD7/Gal4 AD-*β*-catenin, Gal4 BD-PDZD7/Gal4 AD-AMOT, or Gal4 BD-PDZD7/Gal4 AD-CADM1 were plated on nonselective (−Leu/Trp) and selective (−Leu/Trp/His) plates with the presence of 2.5 mM 3-AT and incubated at 30°C for 3 days.

Three candidate proteins *β*-catenin, AMOT, and CADM1 were picked to test the specificity of the interactions by introducing the bait plasmid and prey plasmids back to the reporter yeast strain AH109. Gal4 BD-PDZD7, Gal4 AD-*β*-catenin, Gal4 AD-AMOT, or Gal4 AD-CADM1 alone did not activate the reporter gene *HIS3*. However, when Gal4 BD-PDZD7 was present, Gal4 AD-*β*-catenin, Gal4 AD-AMOT, or Gal4 AD-CADM1 activated *HIS3* expression, suggesting that these proteins specifically interact with PDZD7 (Figures 1(a)–1(c)).

### 3.2. PDZD7 Colocalizes with *β*-Catenin, AMOT, and CADM1 When Overexpressed in COS-7 Cells

**Figure 2:**
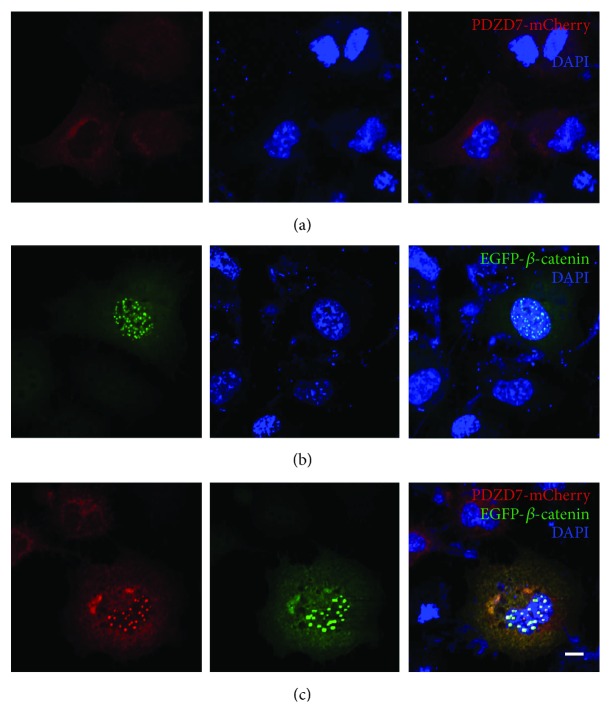
Colocalization of PDZD7 with *β*-catenin in COS-7 cells. Expression vectors were transfected into COS-7 cells, and the subcellular localization of target proteins was determined using confocal microscopy. (a) PDZD7-mCherry localized in the cytoplasm as well as on the plasma membrane. (b) EGFP-*β*-catenin mainly localized in the nuclei in a punctate pattern. (c) When expressed together, PDZD7-mCherry colocalized with EGFP-*β*-catenin in the nuclei. Scale bar: 10 *μ*m.

Next, we examined the subcellular localization of PDZD7 in the presence of these candidate binding partners in cultured cells. When overexpressed in COS-7 cells, PDZD7-mCherry localized in the cytoplasm as well as on the plasma membrane (Figure 2(a)), whereas EGFP-*β*-catenin mainly localized in the nuclei in a punctate pattern (Figure 2(b)). Noticeably, when expressed together with EGFP-*β*-catenin, PDZD7-mCherry translocated into the nuclei and colocalized with EGFP-*β*-catenin (Figure 2(c)), in consistent with the potential interaction between these two proteins.

**Figure 3:**
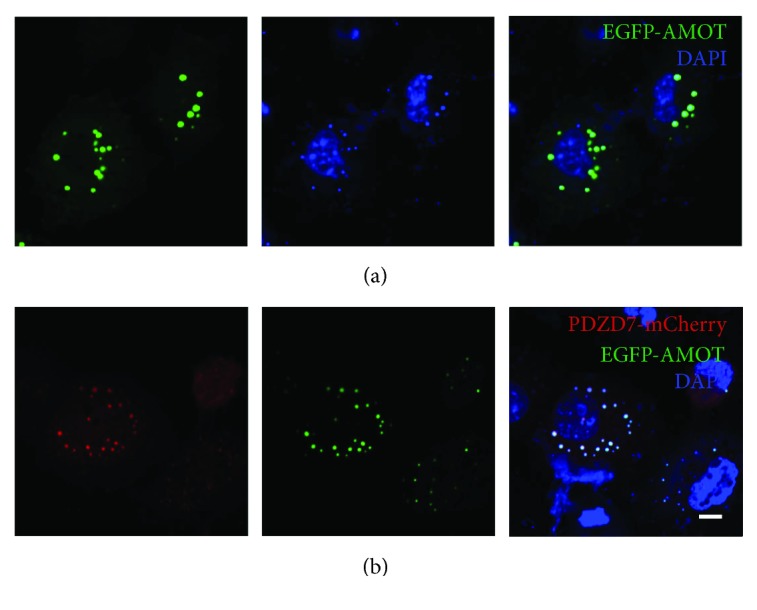
Colocalization of PDZD7 with AMOT in COS-7 cells. Expression vectors were transfected into COS-7 cells, and the subcellular localization of target proteins was determined using confocal microscopy. (a) EGFP-AMOT localized as perinuclear aggregates when expressed alone in COS-7 cells. (b) When expressed together, PDZD7-mCherry colocalized with EGFP-AMOT. Scale bar: 10 *μ*m.

**Figure 4:**
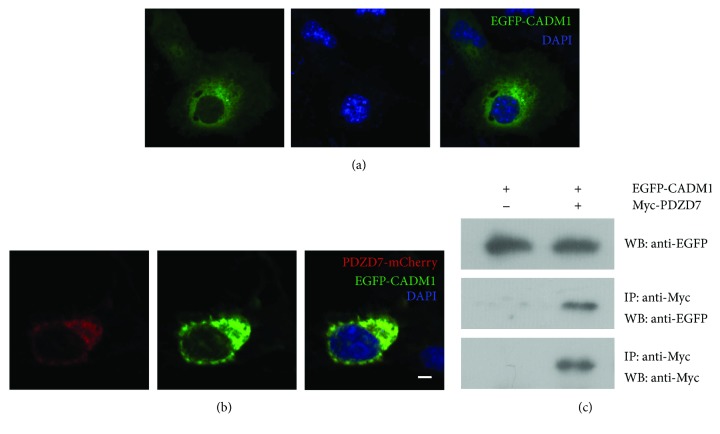
Interaction of PDZD7 with CADM1 in transfected cells. Expression vectors were transfected into COS-7 cells, and the subcellular localization of target proteins was determined using confocal microscopy. (a) EGFP-CADM1 localized in the cytoplasm of COS-7 cells. (b) When expressed together, PDZD7-mCherry colocalized with EGFP-CADM1. Scale bar: 10 *μ*m. (c) Western blots showed that CADM1 was coimmunoprecipitated with PDZD7. Expression vectors were transfected into HEK293T cells to express epitope-tagged PDZD7 and CADM1 proteins, and cell lysates were subjected to immunoprecipitation. IP indicates antibody used for immunoprecipitation, and WB indicates antibody used for detection.

Colocalization was also observed between PDZD7 with AMOT and CADM1. EGFP-AMOT localized as perinuclear aggregates in transfected COS-7 cells (Figure 3(a)). When coexpressed, PDZD7-mCherry colocalized with EGFP-AMOT (Figure 3(b)). Similarly, PDZD7-mCherry colocalized with EGFP-CADM1 in the cytoplasm (Figures 4(a) and 4(b)). Taken together, the colocalization results are consistent with the yeast two-hybrid results, confirming that *β*-catenin, AMOT, and CADM1 are PDZD7-binding partners.

### 3.3. *Cadm1* Expression in the Mouse Inner Ear

**Figure 5:**
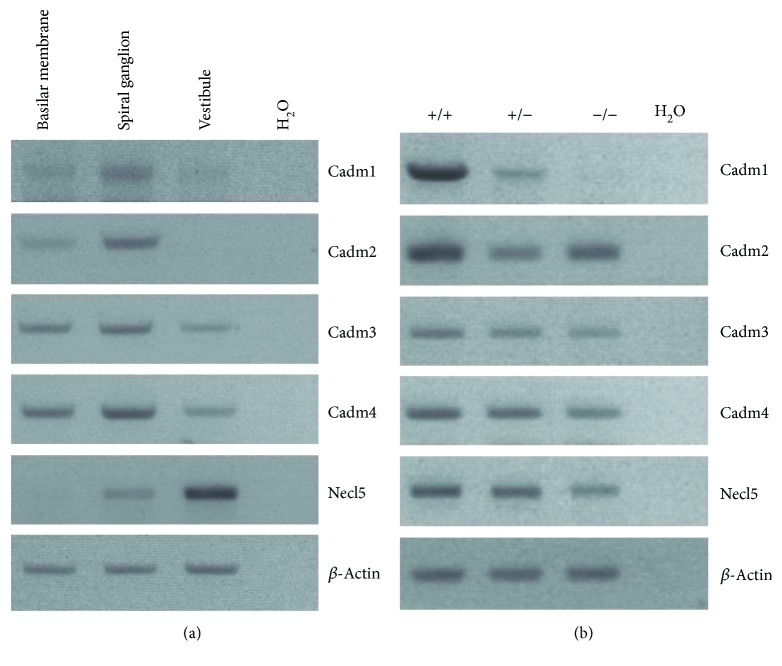
Expression of Necl family members in mouse inner ear. Total RNA from postnatal day 2 (P2) mice was extracted and reverse-transcribed into cDNA, which was then used as PCR template to examine the expression of Necl family members. (a) Expression of Necl family members in basilar membrane, spiral ganglion, and vestibule of wild-type mice was examined through RT-PCR. (b) Expression of Necl family members in the inner ear of *Cadm1* knockout mice was examined through RT-PCR. *β-Actin* was included as internal control.

**Figure 6:**
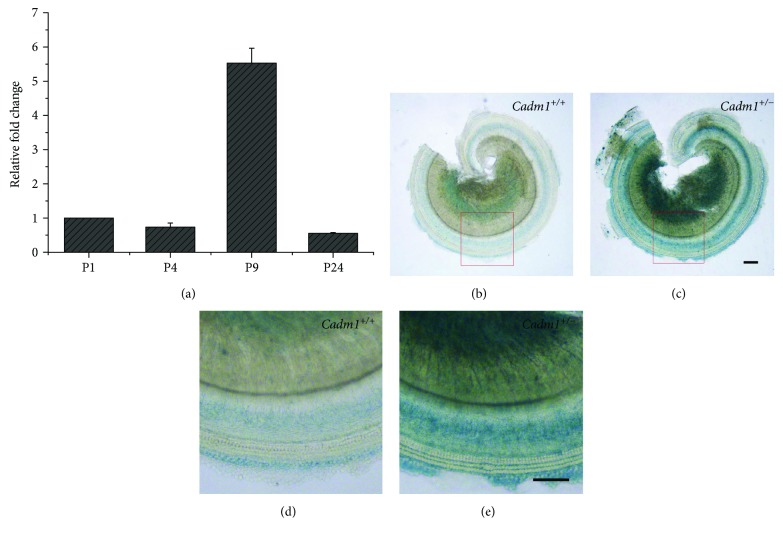
Expression pattern of *Cadm1* in mouse inner ear. (a) Expression of *Cadm1* in different developmental stages of mouse inner ear examined by Q-PCR. The bar graphs show quantification of the PCR results with each value representing the mean ± standard error. (b) LacZ activity in the basilar membrane and spiral ganglion of P7 *Cadm1^+/+^* mice. (c) LacZ activity in the basilar membrane and spiral ganglion of P7 *Cadm1^+/−^* mice. (d) Higher-magnification image from the insect of (b). (e) Higher-magnification image from the insect of (c). Scale bars, 100 *μ*m.

Among the identified candidate PDZD7-binding partners, CADM1 attracted our most attention. The interaction between CADM1 and PDZD7 was further confirmed by co-IP of epitope-tagged proteins (Figures 4(c)). RT-PCR results showed that *Cadm1* is highly expressed in the spiral ganglion and weakly expressed in the basilar membrane (Figure 5(a)). The expression of *Cadm1* in the developing inner ear was examined by performing quantitative real-time PCR (Q-PCR), which showed that *Cadm1* was detected in all developmental stages examined, peaking at around postnatal day 9 (P9) (Figure 6(a)).

The expression pattern of *Cadm1* in the cochlea was further examined using a mouse model whose exon 1 of *Cadm1* gene was replaced by *lacZ* reporter gene cassette [30, 31]. X-gal staining of P7 *Cadm^+/−^* inner ear suggested that *Cadm1* is abundantly expressed in the spiral ganglion. At this stage, the expression of *Cadm1* in the basilar membrane was relatively weak and mainly enriched in supporting cells (Figures 6(b)–6(e)).

### 3.4. *Cadm1* Knockout Mice Have Normal Hearing Threshold

**Figure 7:**
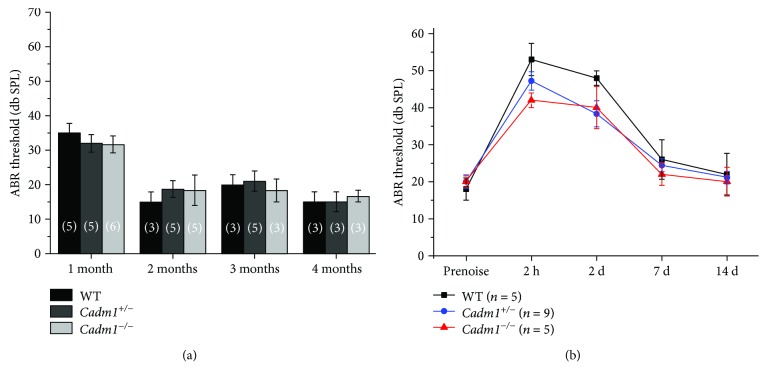
Auditory brainstem response (ABR) measurements show normal auditory function of *Cadm1* knockout mice. (a) ABR thresholds of wild-type, *Cadm1^+/−^*, and *Cadm1^−/−^* mice to click stimuli were measured at different ages as indicated. (b) Wild-type, *Cadm1^+/−^*, and *Cadm1^−/−^* mice at P45 were subjected to 2–8 kHz noise at 96 dB DSL for 2 hours, and ABR thresholds were measured preexposure and at different postexposure time points as indicated. Variance bars indicate standard error. No significant differences were observed between genotypes. The numbers of animals for each group used in the experiments are indicated.

We then evaluated the effect of *Cadm1* disruption on mouse auditory function by performing ABR measurement. The result showed that hearing thresholds of 1-month-old to 4-month-old *Cadm1^−/−^* mice were comparable to those of wild-type or *Cadm1^+/−^* mice, suggesting that CADM1 is not indispensable for hearing transduction (Figure 7(a)). To investigate whether *Cadm1^−/−^* mice show increased acoustic vulnerability, we exposed P45 mice to 2–8 kHz noise at 96 dB SPL for 2 hours. ABR thresholds were measured before and after the noise exposure, which did not reveal any significant difference between *Cadm1^−/−^* and *Cadm1^+/−^* or wild-type mice (Figure 7(b)). Taken together, our results suggested that the auditory function of *Cadm1^−/−^* mice is normal.

The normal hearing threshold of *Cadm1* knockout mice promoted us to look for possible explanations. It has been suggested that the loss of specular protein might be compensated for by its homologous protein(s). As an immunoglobulin- (Ig-) like cell adhesion molecule (CAM), CADM1 belongs to nectin-like molecule (Necl) family, which contains five members (CADM1, CADM2, CADM3, CADM4, and Necl5) [32, 33]. We examined the expression of Necl family members in mouse inner ear by performing RT-PCR. The results showed that all members are expressed in the mouse inner ear (Figure 5(a)), whereas none of them is upregulated in *Cadm1* knockout mice (Figure 5(b)).

## 4. Discussion

*PDZD7* is an important deafness gene, whose mutations contribute to syndromic as well as nonsyndromic hearing loss [17–20]. PDZD7 is a scaffold protein containing three PDZ domains, a HNL domain, and a PR region. Scaffold proteins are important for organizing multiple proteins into protein complex. At present, only a few PDZD7-binding proteins have been reported, including usherin, ADGRV1, whirlin, harmonin, SANS, and MYO7A [17, 18, 21–25]. In this work, we used yeast two-hybrid screening to identify new PDZD7-binding proteins, which will help us to learn more about the role of PDZD7 in hearing transduction.

Among the potential PDZD7-binding partners identified in this work, *β*-catenin is the most frequently encountered one. Wnt/*β*-catenin signaling pathway plays pivotal roles in development, tissue homeostasis, and so on [34]. It has been suggested that Wnt/*β*-catenin signaling regulates proliferation of sensory precursors in the postnatal mouse cochlea [35, 36]. *β*-Catenin could upregulate the expression of Atoh1, a transcription factor that is critical for hair-cell differentiation [37]. Consistently, loss of *β*-catenin inhibited hair-cell differentiation from sensory progenitors [38], whereas forced stabilization of *β*-catenin in supporting cells resulted in proliferation of supporting cells and generation of hair cells [39]. Our data show that PDZD7 interacted with *β*-catenin and that PDZD7 translocated into the nuclei together with *β*-catenin in transfected cells, suggesting a potential role of PDZD7 in regulating *β*-catenin pathway. Further investigation is needed to fully understand the significance and the mechanism of this interaction.

Gelsolin is a calcium-activated actin-binding protein and plays important roles in F-actin severing, capping, and nucleation [40, 41]. It has been shown that gelsolin binds p55 and localizes to the tips of shorter stereocilia of outer hair cells (OHCs) [42]. In mice lacking gelsolin, stereocilia in the apex of the cochlea became long and straggly, suggesting that gelsolin is involved in the regulation of stereocilia elongation [42, 43]. Our data suggested that PDZD7 might interact with gelsolin, hence might play a role in stereocilia development and/or maintenance. Consistent with this hypothesis, OHC stereocilia disorganization has been observed in *Pdzd7* knockout mice [21].

Numb is an evolutionary conserved protein with multiple functions such as asymmetric cell division control, cell fate determination, endocytosis, cell adhesion, cell migration, ubiquitination of specific substrates, and a number of signaling pathways [44]. It has been reported that Numb was expressed in rat cochlear sensory epithelium, and overexpression of Numb upregulated the expression of *Atoh1* in cochlear whole mount cultures [45]. The potential interaction of PDZD7 with Numb raises the possibility that PDZD7 might regulate the function of Numb, which awaits further investigation.

Unlike ADGRV1, *β*-catenin, gelsolin, and Numb, the other PDZD7-binding proteins identified in the present work have not been reported to function in the inner ear. Genes encoding some of the proteins including CADM1, AMOT, Golgin45, and KCTD10 have been detected in mouse cochlea by RNA transcriptome sequencing (SHIELD, https://shield.hms.harvard.edu) [46]. Among these proteins, CADM1 attracted most our attention. CADM1 is an immunoglobulin (Ig) superfamily protein that contains extracellular Ig-like domains, a single transmembrane domain, and a small intracellular C-terminal tail. CADM1 can bind either transhomophilically or transheterophilically with other nectins or Necls [47, 48]. CADM1 plays important roles in modulating synapse development and plasticity, and mutations in *CADM1/Cadm1* gene have been associated with autism spectrum disorder [49–51]. We show here that CADM1 interacts with PDZD7 and *Cadm1* is abundantly expressed in mouse inner ear. However, our data did not reveal any auditory deficit in *Cadm1* knockout mice, suggesting that CADM1 is dispensable for hearing function in mice. Alternatively, other Necl family members might compensate for the loss of CADM1 in the inner ear. Similar scenario has been observed in the neuromuscular junction (NMJ) of *Cadm1* knockout mice, where the loss of CADM1 was compensated for by CADM4 [52].

In conclusion, our present work identified several novel inner ear-expressed PDZD7-binding partners, which will help us to learn more about the role of PDZD7 in hearing. Further investigation is needed to fully understand the biological significance of these interactions.

## Figures and Tables

**Table 1:** Potential PDZD7-binding partners identified from yeast two-hybrid screening. Fragment containing the first two PDZ domains of PDZD7 was used as bait to screen a chicken cochlear cDNA library.

GenBank accession number	Protein	Prey redundancy	PBI
NM_205081	*β*-Catenin	13	-DTDL
XM_015280551	ADGRV1 (VLGR1)	4	-DTHL
NM_204934	Gelsolin	2	-DVDV
XM_004935864	TRIM35	1	-DVPV
XM_015298217	CADM1	1	-EYFI
XM_004940799	Angiomotin (AMOT)	1	-EYLI
XM_416590	Golgin45 (BLZF1)	1	-LIAL
XM_015286934	Numb	1	-EIEL
XM_004945619	KCTD10	2	
XM_015297094	CCDC27	2	
XM_421324	TRIP11 (GMAP210)	1	

## References

[1] Boughman J. A., Vernon M., Shaver K. A. (1983). Usher syndrome: definition and estimate of prevalence from two high-risk populations. *Journal of Chronic Diseases*.

[2] Keats B. J. B., Corey D. P. (1999). The usher syndromes. *American Journal of Medical Genetics*.

[3] Well D., Blanchard S., Kaplan J. (1995). Defective myosin VIIA gene responsible for Usher syndrome type IB. *Nature*.

[4] Eudy J. D., Weston M. D., Yao S. (1998). Mutation of a gene encoding a protein with extracellular matrix motifs in Usher syndrome type IIa. *Science*.

[5] Bitner-Glindzicz M., Glaser B., Lindley K. J. (2000). A recessive contiguous gene deletion causing infantile hyperinsulinism, enteropathy and deafness identifies the Usher type 1C gene. *Nature Genetics*.

[6] Verpy E., Leibovici M., Zwaenepoel I. (2000). A defect in harmonin, a PDZ domain-containing protein expressed in the inner ear sensory hair cells, underlies Usher syndrome type 1C. *Nature Genetics*.

[7] Ahmed Z. M., Riazuddin S., Bernstein S. L. (2001). Mutations of the protocadherin gene *PCDH15* cause Usher syndrome type 1F. *The American Journal of Human Genetics*.

[8] Alagramam K. N., Yuan H., Kuehn M. H. (2001). Mutations in the novel protocadherin *PCDH15* cause Usher syndrome type 1F. *Human Molecular Genetics*.

[9] Bolz H., von Brederlow B., Ramírez A. (2001). Mutation of *CDH23*, encoding a new member of the cadherin gene family, causes Usher syndrome type 1D. *Nature Genetics*.

[10] Bork J. M., Peters L. M., Riazuddin S. (2001). Usher syndrome 1D and nonsyndromic autosomal recessive deafness DFNB12 are caused by allelic mutations of the novel cadherin-like gene *CDH23*. *The American Journal of Human Genetics*.

[11] Joensuu T., Hämäläinen R., Yuan B. (2001). Mutations in a novel gene with transmembrane domains underlie Usher syndrome type 3. *The American Journal of Human Genetics*.

[12] Weil D., el-Amraoui A., Masmoudi S. (2003). Usher syndrome type I G (USH1G) is caused by mutations in the gene encoding SANS, a protein that associates with the USH1C protein, harmonin. *Human Molecular Genetics*.

[13] Weston M. D., Luijendijk M. W. J., Humphrey K. D., Möller C., Kimberling W. J. (2004). Mutations in the *VLGR1* gene implicate G-protein signaling in the pathogenesis of Usher syndrome type II. *The American Journal of Human Genetics*.

[14] Ebermann I., Scholl H. P. N., Charbel Issa P. (2007). A novel gene for Usher syndrome type 2: mutations in the long isoform of whirlin are associated with retinitis pigmentosa and sensorineural hearing loss. *Human Genetics*.

[15] Riazuddin S., Belyantseva I. A., Giese A. P. J. (2012). Alterations of the CIB2 calcium- and integrin-binding protein cause Usher syndrome type 1J and nonsyndromic deafness DFNB48. *Nature Genetics*.

[16] Mathur P., Yang J. (2015). Usher syndrome: hearing loss, retinal degeneration and associated abnormalities. *Biochimica et Biophysica Acta (BBA) - Molecular Basis of Disease*.

[17] Ebermann I., Phillips J. B., Liebau M. C. (2010). *PDZD7* is a modifier of retinal disease and a contributor to digenic Usher syndrome. *The Journal of Clinical Investigation*.

[18] Schneider E., Märker T., Daser A. (2009). Homozygous disruption of *PDZD7* by reciprocal translocation in a consanguineous family: a new member of the Usher syndrome protein interactome causing congenital hearing impairment. *Human Molecular Genetics*.

[19] Booth K. T., Azaiez H., Kahrizi K. (2015). *PDZD7* and hearing loss: more than just a modifier. *American Journal of Medical Genetics Part A*.

[20] Vona B., Lechno S., Hofrichter M. A. H. (2016). Confirmation of *PDZD7* as a nonsyndromic hearing loss gene. *Ear and Hearing*.

[21] Zou J., Zheng T., Ren C. (2014). Deletion of *PDZD7* disrupts the Usher syndrome type 2 protein complex in cochlear hair cells and causes hearing loss in mice. *Human Molecular Genetics*.

[22] Grati M., Shin J. B., Weston M. D. (2012). Localization of PDZD7 to the stereocilia ankle-link associates this scaffolding protein with the Usher syndrome protein network. *The Journal of Neuroscience*.

[23] Chen Q., Zou J., Shen Z., Zhang W., Yang J. (2014). Whirlin and PDZ domain-containing 7 (PDZD7) proteins are both required to form the quaternary protein complex associated with Usher syndrome type 2. *The Journal of Biological Chemistry*.

[24] Morgan C. P., Krey J. F., Grati M.'. (2016). PDZD7-MYO7A complex identified in enriched stereocilia membranes. *eLife*.

[25] Zou J., Chen Q., Almishaal A. (2017). The roles of USH1 proteins and PDZ domain-containing USH proteins in USH2 complex integrity in cochlear hair cells. *Human Molecular Genetics*.

[26] Nie H., Liu Y., Yin X. (2016). Plasma membrane targeting of protocadherin 15 is regulated by the golgi-associated chaperone protein PIST. *Neural Plasticity*.

[27] Liu C., Zhai X., Zhao B., Wang Y., Xu Z. (2017). Cyclin I-like (CCNI2) is a cyclin-dependent kinase 5 (CDK5) activator and is involved in cell cycle regulation. *Scientific Reports*.

[28] Liu Y., Nie H., Liu C. (2017). Angulin proteins ILDR1 and ILDR2 regulate alternative pre-mRNA splicing through binding to splicing factors TRA2A, TRA2B, or SRSF1. *Scientific Reports*.

[29] Heller S., Sheane C. A., Javed Z., Hudspeth A. J. (1998). Molecular markers for cell types of the inner ear and candidate genes for hearing disorders. *Proceedings of the National Academy of Sciences of the United States of America*.

[30] Fujita E., Kouroku Y., Ozeki S. (2006). Oligo-astheno-teratozoospermia in mice lacking RA175/TSLC1/SynCAM/IGSF4A, a cell adhesion molecule in the immunoglobulin superfamily. *Molecular and Cellular Biology*.

[31] Fujita E., Tanabe Y., Hirose T. (2007). Loss of partitioning-defective-3/isotype-specific interacting protein (par-3/ASIP) in the elongating spermatid of RA175 (IGSF4A/SynCAM)-deficient mice. *The American Journal of Pathology*.

[32] Takai Y., Miyoshi J., Ikeda W., Ogita H. (2008). Nectins and nectin-like molecules: roles in contact inhibition of cell movement and proliferation. *Nature Reviews Molecular Cell Biology*.

[33] Mandai K., Rikitake Y., Mori M., Takai Y. (2015). Nectins and nectin-like molecules in development and disease. *Current Topics in Developmental Biology*.

[34] Logan C. Y., Nusse R. (2004). The Wnt signaling pathway in development and disease. *Annual Review of Cell and Developmental Biology*.

[35] Chai R., Kuo B., Wang T. (2012). Wnt signaling induces proliferation of sensory precursors in the postnatal mouse cochlea. *Proceedings of the National Academy of Sciences of the United States of America*.

[36] Shi F., Kempfle J. S., Edge A. S. B. (2012). Wnt-responsive Lgr5-expressing stem cells are hair cell progenitors in the cochlea. *The Journal of Neuroscience*.

[37] Shi F., Cheng Y. F., Wang X. L., Edge A. S. B. (2010). *β*-catenin up-regulates *Atoh1* expression in neural progenitor cells by interaction with an *Atoh1* 3′ enhancer. *The Journal of Biological Chemistry*.

[38] Shi F., Hu L., Jacques B. E., Mulvaney J. F., Dabdoub A., Edge A. S. B. (2014). *β*-Catenin is required for hair-cell differentiation in the cochlea. *The Journal of Neuroscience*.

[39] Shi F., Hu L., Edge A. S. B. (2013). Generation of hair cells in neonatal mice by *β*-catenin overexpression in Lgr5-positive cochlear progenitors. *Proceedings of the National Academy of Sciences of the United States of America*.

[40] Spinardi L., Witke W. (2007). Gelsolin and diseases. *Subcellular Biochemistry*.

[41] Li G. H., Arora P. D., Chen Y., McCulloch C. A., Liu P. (2012). Multifunctional roles of gelsolin in health and diseases. *Medicinal Research Reviews*.

[42] Mburu P., Romero M. R., Hilton H. (2010). Gelsolin plays a role in the actin polymerization complex of hair cell stereocilia. *PLoS One*.

[43] Olt J., Mburu P., Johnson S. L. (2014). The actin-binding proteins eps8 and gelsolin have complementary roles in regulating the growth and stability of mechanosensory hair bundles of mammalian cochlear outer hair cells. *PLoS One*.

[44] Gulino A., Di Marcotullio L., Screpanti I. (2010). The multiple functions of Numb. *Experimental Cell Research*.

[45] Gao Z., Chi F. L., Huang Y. B., Yang J. M., Cong N., Li W. (2011). Expression of Numb and Numb-like in the development of mammalian auditory sensory epithelium. *Neuroreport*.

[46] Shen J., Scheffer D. I., Kwan K. Y., Corey D. P. (2015). SHIELD: an integrative gene expression database for inner ear research. *Database*.

[47] Chan C. J., Andrews D. M., Smyth M. J. (2012). Receptors that interact with nectin and nectin-like proteins in the immunosurveillance and immunotherapy of cancer. *Current Opinion in Immunology*.

[48] Rikitake Y., Mandai K., Takai Y. (2012). The role of nectins in different types of cell–cell adhesion. *Journal of Cell Science*.

[49] Zhiling Y., Fujita E., Tanabe Y., Yamagata T., Momoi T., Momoi M. Y. (2008). Mutations in the gene encoding CADM1 are associated with autism spectrum disorder. *Biochemical and Biophysical Research Communications*.

[50] Fujita E., Dai H., Tanabe Y. (2010). Autism spectrum disorder is related to endoplasmic reticulum stress induced by mutations in the synaptic cell adhesion molecule, CADM1. *Cell Death & Disease*.

[51] Takayanagi Y., Fujita E., Yu Z. (2010). Impairment of social and emotional behaviors in Cadm1-knockout mice. *Biochemical and Biophysical Research Communications*.

[52] Tanabe Y., Fujita E., Hayashi Y. K. (2013). Synaptic adhesion molecules in Cadm family at the neuromuscular junction. *Cell Biology International*.

